# A survey of *ex vivo/in vitro* transduction efficiency of mammalian primary cells and cell lines with Nine natural adeno-associated virus (AAV1-9) and one engineered adeno-associated virus serotype

**DOI:** 10.1186/1743-422X-10-74

**Published:** 2013-03-06

**Authors:** Brian L Ellis, Matthew L Hirsch, Jenny C Barker, Jon P Connelly, Robert J Steininger, Matthew H Porteus

**Affiliations:** 1 Department of Biochemistry, University of Texas Southwestern Medical Center, Dallas, TX, USA; 2 Gene Therapy Center, University of North Carolina at Chapel Hill, Chapel Hill, NC, USA; 3 Department of Ophthalmology, University of North Carolina at Chapel Hill, Chapel Hill, NC, USA; 4 Department of Pharmacology, Green Center for Systems Biology, Simmons Cancer Center, University of Texas Southwestern Medical Center, Dallas, TX, USA; 5 Department of Pediatrics, University of Texas Southwestern Medical Center, Dallas, TX, 75390-9148, USA

**Keywords:** AAV, Serotypes, Adeno-associated virus, Gene therapy, Tropism, Primary cells, Progenitor cells, Cell lines, Transduction, *ex vivo*

## Abstract

**Background:**

The ability to deliver a gene of interest into a specific cell type is an essential aspect of biomedical research. Viruses can be a useful tool for this delivery, particularly in difficult to transfect cell types. Adeno-associated virus (AAV) is a useful gene transfer vector because of its ability to mediate efficient gene transduction in numerous dividing and quiescent cell types, without inducing any known pathogenicity. There are now a number of natural for that designed AAV serotypes that each has a differential ability to infect a variety of cell types. Although transduction studies have been completed, the bulk of the studies have been done *in vivo*, and there has never been a comprehensive study of transduction *ex vivo/in vitro*.

**Methods:**

Each cell type was infected with each serotype at a multiplicity of infection of 100,000 viral genomes/cell and transduction was analyzed by flow cytometry + .

**Results:**

We found that AAV1 and AAV6 have the greatest ability to transduce a wide range of cell types, however, for particular cell types, there are specific serotypes that provide optimal transduction.

**Conclusions:**

In this work, we describe the transduction efficiency of ten different AAV serotypes in thirty-four different mammalian cell lines and primary cell types. Although these results may not be universal due to numerous factors such as, culture conditions and/ or cell growth rates and cell heterogeneity, these results provide an important and unique resource for investigators who use AAV as an *ex vivo* gene delivery vector or who work with cells that are difficult to transfect.

## Background

A fundamental technique in biomedical research is to deliver a gene of interest (transgene) into a cell in order to alter its behavior. While transgene delivery can be achieved by a number of different transfection strategies, such as chemical, lipid, or electroporation based methods, there are many cell types that are not efficiently transfected in these ways (for example primary T-cells, cardiomyocytes, and primary hematopoietic stem cells). Viral vectors have become an important resource to overcome these barriers to gene transfer. There are a number of different viral vectors that have been used for gene transfer, including retroviruses, lentiviruses, and adenovirus, but one of the most utilized viral vectors has been recombinant adeno-associated virus (AAV). While AAV serotype 2 (AAV2) has been the most widely used AAV vector, there are now multiple natural and designed AAV capsid variants, each of which has a different tropism for different cell types [1,2]. Thus, AAV is a generally useful vector for gene transfer in a wide range of cell types.

**Figure 1:**
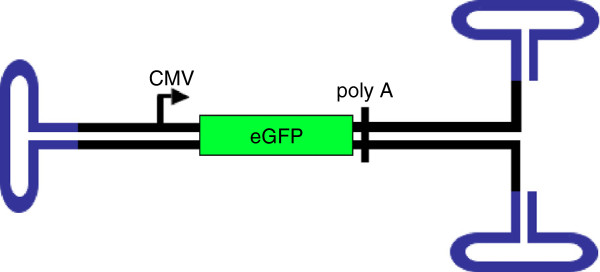
**A schematic representation of the self-complimentary AAV (scAAV) genome.** We constructed a scAAV genome (double black lines) that included an eGFP reporter gene (in green) driven by the CMV promoter (black arrow) that allowed for efficient and quantitative analysis of transduction. This construct also included a poly A sequence (black line) and inverted terminal repeats (ITRs) in blue)).

AAV is a small, non-enveloped virus that packages both negative and positive polarity single-stranded DNA. AAV is a member of the Parvoviridae family and requires a helper virus, such as adenovirus or herpesvirus, for a productive infection. The wild-type genome is 4.7 kb and contains two major open reading frames (ORFs) that include the Rep gene and Cap gene. In addition, a third ORF was recently shown to exist [3]. When AAV is used as a gene transfer vector, the endogenous genes are removed and replaced by an expression cassette for the gene of interest. One of the barriers to efficient expression of the transgene is the conversion of the single-strand AAV (ssAAV) genome into a duplexed single DNA molecule [4]. The limitation in transgene expression from ssAAV vectors has been improved by the development of self-complementary AAV (scAAV) vectors in which the single-stranded AAV genome self-hybridizes to form duplex DNA (Figure 1). scAAV vectors have shown earlier onset of transgene expression and overall higher transduction efficiencies than ssAAV vectors [4,5].

The ability to transduce different cell types is primarily determined by the AAV protein capsid [1]. The different capsids bind to different cellular receptors and this binding mediates entry into the cell. The primary receptor for AAV2 and AAV3 is heparan sulfate-proteoglycan [6]. Integrin α5β5, integrin α5β1, hepatocyte growth factor receptor (c-Met), and CD9 have also been described previously as potential co-receptors for AAV2 [7-10]. The fibroblast growth factor receptor-1 is a co-receptor for both AAV2 and AAV3 [11] and the 37/67-kDa laminin receptor is a co-receptor for AAV2, AAV3, AAV8 and AAV9 [12,13]. The primary receptor for AAV1, AAV4, and AAV5 is O-linked sialic acid, while the primary receptor for AAV6 is N-linked sialic acid [14-17]. The platelet derived growth factor receptor is a co-receptor for AAV5 [18]. The consequence of the different cellular receptors for capsid binding is that each of these natural AAV serotypes transduces a different range of cell types. The ability of different AAV serotypes to transduce different cell types has been previously reported, but most of these studies have been done *in vivo*, and they report on the effectiveness of tissue type transduction rather than cell type transduction [2]. For example, a broad study of AAV serotypes 1-9 has been done in mice [19]. However, a complete *ex vivo/in vitro* study of transduction efficiency is lacking. In this work, we performed an extensive survey where thirty-four different mammalian cell types were transduced with ten different AAV serotypes *ex vivo/in vitro*. This collective data provides an important and unique resource to the researchers interested in gene delivery *ex vivo* and in cultured cell lines. Our data clearly demonstrate that there are clear qualitative differences for the ability of different serotypes to transduce different sub-types giving general guidance on the best serotypes to use and that *a priori* prediction is not always possible. Transduction variability could be high, particularly when the infection efficiency is low, and suggest that the data should be generally viewed in 7 broad categories: 1: 0%, 2: >0-1%, 3: 1-10%, 4: 10-30%, 5: 30-60%, 6: 60-80%, 7: 80-100%. Moreover, these categories should not be viewed as rigid as it is likely that transduction of 8%, for example, is not necessarily different than 12%.

## Results and discussion

**Figure 2:**
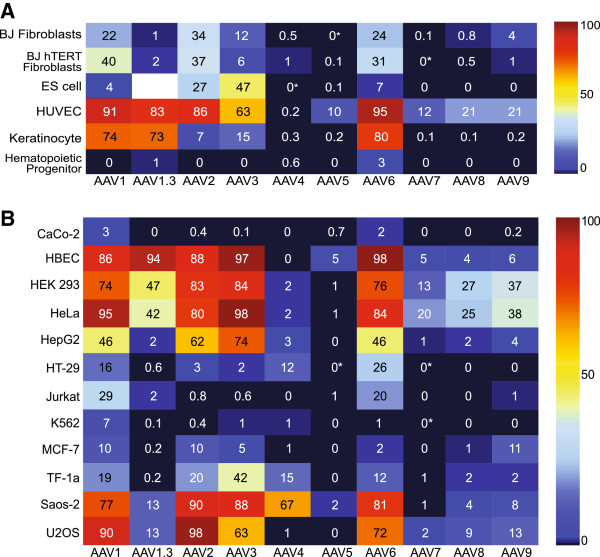
**scAAV transduction of human primary and immortalized cells. A)** Human primary cells and **B)** human immortalized cell lines were transduced with eGFP scAAV at a multiplicity of infection (MOI) of 100,000 viral genomes (vg)/ cell. The cells were analyzed by flow cytometry at 48 hours post-infection for the percentage that were GFP positive. The number in the box is the actual percentage of GFP positive cells with that serotype. * = Transduction less than 0.01% but greater than 0.0%.

**Figure 3:**
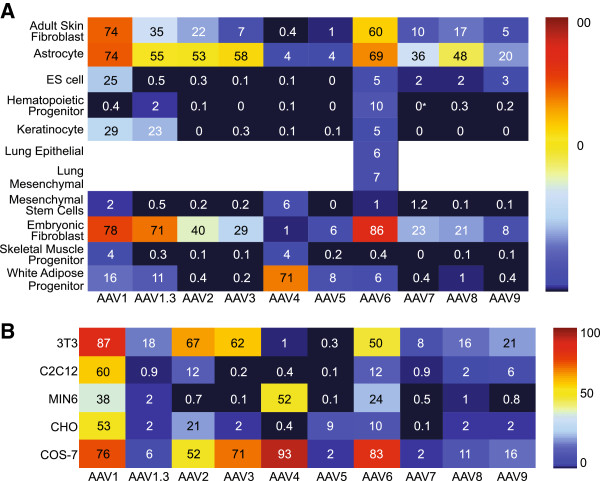
**scAAV transduction of murine primary cells and murine, hamster, and monkey immortalized cells. A)** Murine primary cells and **B)** murine, hamster, and monkey immortalized cell lines were transduced with eGFP scAAV at a multiplicity of infection (MOI) of 100,000 vg/ cell. The cells were analyzed by flow cytometry at 48 hours post-infection for the percentage that were GFP positive. The number in the box is the actual percentage of GFP positive cells with that serotype.

To analyze the tropism of nine different natural AAV serotypes (1-9) and one engineered serotype (1.3) (a hybrid of AAV1 and AAV6), we used scAAV vectors that expressed eGFP from the CMV promoter (Figure 1). Even though ssAAV has a larger cloning capacity than scAAV, we chose scAAV because of the overall improved transgene expression of its vectors compared to ssAAV vectors as this report was intended to be a straightforward capsid comparison. Because some cells have been reported to be refractory to AAV transduction, we wanted to use the most efficient genome technology helping to reduce the possibility that timing and amount of transgene expression would bias the results. We selected eGFP as a transgene because of the ease of quantitating transgene product fluorescence by flow cytometry and because live cultures could be analyzed by microscopy. We infected all cell lines at a constant multiplicity of infection [MOI (defined here as vector genomes per cell)] of 100,000 vector genomes/cell and analyzed for eGFP expression two days after infection. Furthermore, we repeated the infections at an MOI of 10,000 and saw the same trends, though a lower percentage of GFP + cells (data not shown). Although, MOIs of 10,000 or 100,000 in some cases might be considered high, MOIs of 10,000 and up to 500,000 have been used for gene targeting [20,21], and importantly, it ensures that if a cell was not transduced it was not because too low of an MOI was tested. The results are presented as heat-maps; higher transduction efficiencies (measured as %GFP + cells) are displayed in red, and lower transduction efficiencies are in blue. The actual transduction efficiency is given as a percentage. A complete list of the cells transduced in both Figures 2 and 3 are presented in Table 1 and a description of the isolation of the primary cells are listed in the Materials and Methods section.

**Table 1:** Cell types and description


**Human Primary Cells**	**Description**
BJ Fibroblasts	Foreskin fibroblasts
BJ hTERT Fibroblasts	Foreskin fibroblasts retrovirally infected with hTERT
ES cell	Embryonic stem cells
HUVEC	Human umbilical cord vein endothelial cells
Karatinocytes	Keratinocytes
Hematopoietic Progenitor	CD34+ umbilical cord cells
**Human Cell Lines**	**Description**
CaCo-2	Epithelial colorectal adenocarcinoma cells
HBEC	Human bronchial epithelial cells
HEK 293	Human embryonic kidney cells
HeLa	Cervical cancer cells
HepG2	Hepatocellular carcinoma
HT29	Colon adenocarcinoma grade II cells
Jurkat	immortalized line of T lymphocyte cells
K562	Myelogenous leukemia cells
MCF-7	Breast cancer cells
TF1a	Erthroleukemic cells
Saos-2	Osteosarcoma cells
U20S	Osteosarcoma cells
**Mouse Primary Cells**	**Description**
Adult Skin Fibroblast	Murine adult skin fibroblasts (MAFs)
Astrocytes	Astrocytes
ES cell	Embryonic stem cells
Hematopoietic Progenitor	cKit+, Sca+, Lin- hematopoietic cells
Keratinocytes	Keratinocytes
Lung Epithelial	Epithelial cells
Lung Mesenchymal	Mesenchymal cells
Mesenchymal Stem Cells	Mesenchymal stem cells
Embryonic Fibroblast	Murine embryonic fibroblasts (MEFs)
Skeletal Muscle Progenitor	Skeletal muscle progenitor cells
White Adipose Progenitor	White adipose progenitor cells
**Mouse Cell Lines**	**Description**
3 T3	Heterogeneous embryonic mouse cells
C2C12	Myoblast cells
MIN6	Pancreatic beta cells
**Other Cell Lines**	**Description**
CHO	Chinese hamster ovary cells
COS-7	African green monkey kidney fibroblasts

Listed are the cell types transduced by AAV1-9 and AAV1.3 in Figures 2 and 3. The Materials and Methods section list a more detailed description of the primary cell isolations.

### Transduction of human primary cells

We evaluated ten different AAV serotypes for their ability to transduce six different purified primary human cell types: BJ fibroblasts, BJ hTERT fibroblasts, embryonic stem cells (ES), human umbilical cord vein endothelial cells (HUVEC), human keratinocytes, and human hematopoietic progenitor cells (Figure 2a). To avoid heterologous mixtures of cells, the primary cell types were either isolated, as described previously (see Materials and Methods), or purchased as purified cells. At 48 hours post infection, we found based on %GFP + cells, that AAV1, 2 and 6 best transduced human fibroblasts, AAV3 was most efficient for human ES cells, AAV1, 1.3, 2, and 6 showed the highest transduction for HUVECs, and AAV1, 1.3 and 6 best transduced keratinocytes. We found none of these serotypes efficiently transduced human hematopoietic progenitor cells (purified CD34+ cells). We note the BJ fibroblasts, BJ hTERT fibroblasts, ES cells, and HUVEC cells are not freshly isolated cells. However, we categorize them as primary cells here because they are not transformed and show the same properties as freshly isolated cells.

### Transduction of human cell lines

In Figure 2b, we report our results for the transduction of twelve different human derived cell lines 48 hours post infection based on %GFP+. We found that Caco-2 (an epithelial colorectal cell type) and K562 cells (a hematopoietic derived cell line) were not efficiently transduced by any of the AAV serotypes, although 20% of TF1-α cells (a different hematopoietic cell line) were GFP + using AAV2. The lack of transduction of K562 cells cannot be explained by the lack of expression from a CMV promoter, as strong CMV mediated transgene expression is obtained after transfection of K562 cells (data not shown). All of the remaining cell types were transduced to at least 11%, and most were transduced at much higher efficiencies. Overall, AAV1 and AAV6 were two of the best serotypes for efficient transduction of human cell lines, while AAV2 and AAV3 were also broadly effective in transduction of human cell lines (Figure 2b).

### Transduction of murine primary cells

We transduced nine different primary murine cell types (adult skin fibroblasts, astrocytes, ES cells, hematopoietic progenitors, keratinocytes, mesenchymal stem cells, murine embryonic fibroblasts (MEF), skeletal muscle progenitor, and white adipose progenitor cells) with the ten different AAV serotypes. We also transduced two different primary murine cell types (murine lung epithelial and lung mesenchymal cells) with AAV6. We then evaluated %GFP + at 48 hours post infection (Figure 3a). All of these murine primary cells were either isolated, as described previously (see Materials and Methods), or purchased as purified cells, avoiding heterologous mixtures of cells. We found that none of the serotypes efficiently transduced mesenchymal stem cells or skeletal muscle progenitors. We showed that AAV6 was the best serotype for transducing hematopoietic progenitor cells, but only to a level of 10%, a relatively low percentage (Figure 3a, row 4). In contrast to most other cell types tested, we found that AAV4 infects white adipose progenitor cells exceptionally well, especially in comparison to all other serotypes (Figure 3a, row 11). We saw that murine ES cells transduce well with AAV1 (Figure 3a, row 3, 25%). This also fits with the data the McWhir group showed using AAV2, 4, and 5 that mES cells were not transduced efficiently [22]. For the remaining cell types, at least one AAV serotype was efficient in mediating transduction. In general, AAV1 and AAV6 were two of the best serotypes for transduction of mouse primary cells under the conditions tested.

### Transduction of monkey, hamster, and mouse cell lines

We transduced five different mammalian cell lines, including murine 3 T3 cells, murine C2C12 cells, murine MIN6 cells, Chinese hamster ovary cells (CHO), and monkey COS-7 cells with the ten different AAV serotypes and evaluated %GFP + at 48 hours post infection (Figure 3b). Every cell line we tested had at least one serotype that transduced cells with >50% transduction efficiency (Figure 3b). In some cases our results did not match perfectly with previous results [23] (for example AAV1, AAV2, AAV5 on C2C12 cells; AAV1, AAV2, AAV5 on CHOK1 cells). With the exception of AAV5 on C2C12 cells, the data presented here reasonably reflect previous data [23] when viewing the panel qualitatively, and the differences in culture conditions and MOI (10,000 or 100,000 vs. 300 vg/cell) could account for the variability. Another discrepancy from our data [24] showed that AAV2 transduced COS cells better than AAV1. Although the difference is dramatic, it could possibly be explained by changes in viral preparation, culture conditions, and the detection system used (hFIX expression vs. GFP expression). Overall, we found that AAV1 and AAV6 are two of the best serotypes to infect cell lines of mouse, hamster, and monkey origin under the conditions tested; however, the particular cell type must be considered, as other serotypes were more efficient in some cases.

## Conclusion

Expansion in the number of AAV serotypes, both through the identification of novel natural serotypes and new, engineered serotypes [16,23,25-35], has resulted in improved gene transfer to specific cell types *in vivo*. There have been several publications reviewing the preferred *in vivo* tropism of these new serotypes [2,36]. However *ex vivo/in vitro* data is lacking. Alternative uses of AAV include using this viral vector *ex vivo* as a method of gene transfer into specific cell types. These transduced cells could then be studied directly or used for cell-based gene therapy, whereby AAV transduction would occur and the modified cells would then be transplanted.

Another potential use of AAV vector transduction *ex vivo* would be to use AAV vectors to stimulate gene targeting by homologous recombination, to precisely modify the genome of the cells to be transplanted. This modification could be done through gene targeting directly by AAV [37-40] or in combination with the induction of a site-specific double-strand break. These site-specific double-strand breaks could be induced by a homing endonuclease [21,41,42], by zinc finger nucleases [43], or by some other nuclease, like TAL effector nucleases (TALENs) [44-47]. An important aspect to using AAV in this manner is to determine the best serotype to transduce specific cell types *ex vivo,* where there is no basement membrane or extracellular matrix. In fact, we have already reported the ability of a single AAV6 vector to deliver both zinc-finger nucleases as well as a donor repair substrate, to stimulate gene targeting [20].

In regards to stem cells, it is intriguing that many progenitor cells did not transduce well (i.e. see lots of dark blue in the heat maps). Perhaps the cells have evolved the ability to avoid transduction as a way to protect themselves from changes to the cell, in particular the DNA. However, there certainly were stem cells that were transduced well by various AAV serotypes. This again points to the utility of this study, as certain serotypes were good for transduction in some stem cells and bad in others and although AAV1 and AAV6 were good or the best at transduction in many stem cells, there were some stem cells that transduced poorly with AAV1 and AAV6.

In this work we provide a broad survey that examines the ability of ten different AAV serotypes to infect thirty-four different cell types *ex vivo/in vitro.* In general, we demonstrate that AAV1 and AAV6 have the greatest ability to transduce a wide range of cell types. We found, however, that for particular cell types there are specific serotypes, which provide optimal transduction. (For example, AAV4 is the optimal serotype for transducing murine adipose progenitor cells.) We also found that there are certain primary cell types, such as human hematopoietic progenitor cells, that were not efficiently transduced by any of the ten different serotypes. It is possible that the lack of measured transduction in these cell types is because the CMV promoter is relatively weak in these cells. It is not likely, however, that in these cases the cells were overloaded with uptake and processing of virions because when a 10-fold lower MOI was used, lower transduction efficiency was seen in every case (data not shown). Although, different growth conditions were used for many of the different cell types, each serotype was used for each cell type in the same conditions, thus providing an internal control for a comparative analysis. However, because a small amount of our data does not perfectly match with previous findings, we suggest that the results presented here should lead investigators to choose a few of the best serotypes for their specific need. Our results demonstrate that there is no simple mechanism to predict which serotype will transduce a particular cell type but does suggest that if one were limited to screening a small number of serotypes that focusing on AAV6, AAV2, and AAV3 would be reasonable as those three serotypes give a broad range of effectiveness across most cell types. In the future, it may be important to further study the transduction of AAV after different purification strategies are used, as it has been shown to affect transduction [48]. Methods to facilitate AAV transduction, such as by the use of proteasome inhibitors [49] or strategies that allow for selection of novel capsids, may help overcome the barrier to transduction that these cells exhibit. However, it is likely, there are factors, unproven as of yet, that serve as major barriers to transduction by AAV. For example, it is possible that the apparent low transduction could be a consequence of AAV vectors inducing apoptosis [50]. In this case, a caspase inhibitor such as Z-VAD-FMK could be used to achieve transduction without cell death. Understanding these unproven barriers to transduction would further improve the utility of AAV as a gene transfer vector for *ex vivo* manipulation of primary cells as well as *in vivo* gene therapy.

In summary, we have performed a survey of the ability of different AAV serotypes to transduce a wide variety of different primary and immortalized cell types. This survey should be a useful and practical resource for investigators as they consider using AAV as a gene transfer vector in their studies.

## Methods

### Ethics statement

All animal work has been conducted according to relevant national and international guidelines. Approval for studies using cells derived from mice was obtained from the UT Southwestern IACUC, APB# 2010-0106.

### AAV production

We thank R.J. Samulski for providing the self-complementary eGFP and the pXR series of plasmids used herein. AAV vector production relied on the triple transfection method described previously [51]. Briefly, cells were transfected with the adenovirus helper plasmid pXX680, pHpa-Trs-SK CMV-eGFP (to generate self-complementary AAV genomes) [5], a plasmid that codes for AAV Rep2, and a specific capsid serotype (pXR series 1-9, corresponding to AAV serotypes 1-9). Three days after HEK 293 cell transfection in a plate format, nuclei were harvested, disrupted, and the lysate was separated by cesium chloride gradient centrifugation [51]. DNase was used during purification. Following an overnight spin at 55,000 rpm, 12 gradient fractions were pulled. To determine the gradient fraction composed of pure scAAV genomes, 10 μL of each fraction was subjected to Southern blotting following alkaline gel electrophoresis as previously described [51]. Fractions containing only scAAV genomes were pooled, dialyzed against 1X PBS, aliquoted and stored at -80 °C until use. Final titer determination was performed after the initial thaw by quantitative PCR using primers specific for the eGFP transgene (forward primer: 5^′^-AGCAGCACGACTTCTTCAAGTC -3^′^; reverse primer: 5^′^-TGTAGTTGTACTCCAGCTTGTGCC-3^′^).

### Human primary cell isolation and culture conditions

BJ fibroblasts and BJ hTERT fibroblasts were a generous gift from Jerry Shay and Woodring Wright and were cultured as previously described [52]. The hES cell line H9 (WA09, XX, Passage 30-35) was cultured on feeder-free fibronectin coated plates with mouse embryonic fibroblast (MEF) conditioned human ES cell medium. MEFs were mitomycin-c inactivated and plated in fibroblast medium, Dulbecco’s Modified Eagle Medium (DMEM) (Invitrogen), supplemented with 10% bovine growth serum (Hyclone, Logan, UT), 2 mM L-glutamine, 100 IU/mL penicillin, and 100 mg/ml streptomycin. 24 hours after attachment, the medium was replaced with hES complete medium (77% DMEM:F12 (Sigma), 20% Knockout SR (Invitrogen), 1% Non-Essential amino acids (Invitrogen), 1% Penicillin/Streptomycin (Invitrogen), 1 mM L-Glutamine (Invitrogen), 0.1 mM beta-mercaptoethanol (Sigma), 4 ng/ml basic Fibroblast Growth Factor (Invitrogen). After 24 hours, the medium was removed, filtered, and used as conditioned medium for human ES cell cultures. Cells were cultured in 5% CO_2_ at 37°C and passaged every 5-6 days to maintain undifferentiated cultures. HUVEC cells (Lonza) were a generous gift from Chieko Mineo and Phil Shaul (University of Texas Southwestern Medical Center in Dallas (UTSW)) and were cultured in Endothelial Cell Growth Medium-2 (EGM-2) (Lonza) with the EGM-2 BulletKit (Lonza). Keratinocytes (Invitrogen) were cultured in Keratinocyte Serum Free Media (KSFM) + supplement (Invitrogen). The hematopoietic progenitor cells (Lonza) were isolated by CD34+ purification from bone marrow and were cultured in hematopoietic progenitor cell medium (Lonza).

### Human cell lines culture conditions

CaCo-2 cells were a generous gift from Jerry Shay and Woodring Wright (UTSW) and were cultured in DMEM (Media Tech) supplemented with 10% bovine growth serum (Hyclone, Logan, UT), 2 mM L-glutamine, 100 IU/mL penicillin, and 100 mg/ml streptomycin. The cultures were grown in a humidified incubator at 37°C with 5% CO_2_. HBEC3KT cells were a generous gift from John Minna (UTSW) and were cultured as previously described [53]. HeLa cells were cultured in HepG2 cells (ATCC) were cultured in the same way as CaCo-2 cells. HT-29 cells were a generous gift from Jerry Shay and Woodring Wright (UTSW) and were cultured in the same way as CaCo-2 cells. Jurkat cells were a generous gift from Zhijian Chen (UTSW) and were cultured in Roswell Park Memorial Institute 1640 (RPMI) (Media Tech) supplemented with 10% bovine growth serum (Hyclone, Logan, UT), 2 mM L-glutamine, 100 IU/mL penicillin, and 100 mg/ml streptomycin. The cultures were grown in a humidified incubator at 37°C with 5% CO_2_. K562 (ATCC) cells were cultured in the same way as Jurkat cells. MCF-7 cells were a generous gift from Rolf Brekken (UTSW) and were cultured in the same way as CaCo-2 cells. TF1α cells were a generous gift from Saswati Chatterjee (City of Hope) and were cultured in the same way as Jurkat cells. Saos-2 cells (ATCC) were cultured in the same way as CaCo-2 cells. U2OS cells were a generous gift from David Spector (Cold Spring Harbor) and were cultured in the same way as CaCo-2 cells.

### Mouse primary cell isolation and culture conditions

Adult skin fibroblasts, astrocytes, ES cells, and embryonic fibroblasts were isolated and cultured as described previously [54]. The hematopoietic progenitor cells were isolated from six to eight week old mice. Mice were sacrificed and whole bone marrow was flushed from femurs and tibias with IMDM 2% FBS. Next whole bone marrow cells were spun down and resuspended in MACS buffer. CD117+ cells were enriched from whole bone marrow by using MACS magnetic bead separation with CD117+ microbeads (Miltenyi), running cells over a MACs MS + column (Miltenyi), and washing the column three times with MACS buffer. The column was then removed from the magnetic field and cells retained in the column were flushed with MACS buffer using a plunger. The CD117+ were then washed and labeled with antibodies to further enrich for long-term repopulating cells using the following antibodies: non-specific binding of antibodies was blocked by incubating cells with a CD16/32 antibody (eBioscience), followed by labeling cells with Lin- antibodies FITC-CD3e (eBioscience), FITC-CD4 (eBioscience), FITC-CD5 (eBioscience), FITC-CD8a (eBioscience), FITC-CD11b (eBioscience), FITC-CD45R (eBioscience), FITC-Ly-6 G (eBioscience), FITC-Ter119 (eBioscience). An APC-Sca-1 (eBioscience) antibody was used to label Sca-1+ cells. FITC-/ APC + cells (KLS cells) were sorted using a FACsAria flow cytometer (BD Bioscience) and cultured in Stemspan (Stemcell Technologies). Murine keratinocytes were isolated and cultured as described previously [55]. The mesenchymal stem cells were isolated and expanded from 8 week old mice as previously described [56]. Briefly, whole bone marrow was flushed from femurs and tibias and plated in Mesencult Proliferation kit media (Stemcell Technologies) in six well plates at a density of 5E6 cells per mL and cultured as described previously. The skeletal muscle progenitor cells were isolated and cultured as described previously [57] (instruction in the isolation method was generously provided by Amy Wagers at Harvard University). The white adipose progenitor cells were isolated and cultured as described previously [58] (isolation instruction was generously provided by Jon Graff at UTSW).

### Mouse, hamster, and monkey cell lines culture conditions

3 T3 cells were created as previously described [54] and were cultured in the same way as CaCo-2 cells. C2C12 cells were a generous gift from Eric Olson (UTSW) and were cultured in the same way as CaCo-2 cells. MIN6 cells were a generous gift from Melanie Cobb (UTSW) and were cultured as described previously [59]. CHO cells were a generous gift from Ben Chen (UTSW) and were cultured in the same way as CaCo-2 cells. COS-7 cells were a generous gift from Carole Mendelson (UTSW) and were cultured in the same way as CaCo-2 cells.

### AAV infection and measurement of GFP positive cells

Experiments for all cell types were performed in this manner, unless otherwise noted. About 10,000 cells per well were plated in 500 μL of media in a 24-well plate. Immediately after plating, the cells were infected with 1 of the 9 AAV serotypes at a multiplicity of infection (MOI) of 100,000 viral genomes per cell (the experiments were repeated using an MOI of 10,000, not shown). At 24 hours, an additional 0.5 mL of media was added to cells (for the cells in a 24 well plate, otherwise the volume was doubled from the original volume). At 48 hours post infection, the cells were harvested and analyzed for GFP expression on a FACS Caliber (Becton-Dickerson, San Jose, CA). For hES cells, AAV transduction experiments were performed 2 days after passage at a time when hES cell colonies were isolated and predominantly monolayers. For murine hematopoietic progenitor cells and murine mesenchymal stem cells, cells were plated at 10,000 cells per well in a 96 well plate and infected, as described above. For white fat progenitor cells, cells were plated at 10,000 cells per well in a 48 well plate and infected as described above. For murine keratinocytes, cells were plated at 20,000 cells per well in a 48 well plate and infected as described above. For murine skeletal muscle progenitors, cells were plated at 2,500 cells per well in a 96 well plate and infected as described above.

Many of the experiments at an MOI of 100,000 were done once, some were done two or three times. Experiments were then repeated at an MOI of 10,000 and the trends were the same (although, as expected, the %GFP were lower). Thus, quantitative values of transduction efficiencies of these serotypes cannot be determined by this data. However, because only one preparation of each serotype was used for all experiments for consistency (and aliquoted and frozen at -80°C), this qualitative data can be used to choose the best serotype(s) for transduction of a particular cell type. Furthermore, the preps of virus made for this survey were first evaluated on 293 cells to ensure that transduction efficiency was comparable to the countless other preps that have been made of these serotypes.

## Abbreviations

AAV: Adeno-associated virus; eGFP: Enhanced green fluorescent protein; PCR: Polymerase chain reaction; PBS: Phosphate buffered saline; HEK: Human embryonic kidney; MOI: Multiplicity of infection; CMV: Cytomegalovirus; TALEN: Tal effector nuclease; ORF: Open reading frame; scAAV: Self complimentary AAV; ssAAV: Single stranded AAV; HUVEC: Human umbilical vein endothelial cell; ES: Embryonic stem; hES: Human embryonic stem; mES: Murine embryonic stem; hTERT: Human telomerase reverse transcriptase; CD: Cluster of differentiation; MEF: Murine embryonic fibroblast; CHO: Chinese hamster ovary; MACS: Magnetic activated cell sorting; FACS: Fluorescent activated cell sorting; vg: Viral genomes.

## Competing interests

The authors have declared that no competing interests exist.

## Authors’ contributions

BE performed infections, isolated primary cells, performed flow cytometry, and wrote the manuscript. JB performed infections, isolated primary cells, and performed flow cytometry. MH made the AAV and helped with drafting/revising the manuscript. JC isolated primary cells. RS created the heat maps. MP conceived the study and helped with drafting/revising the manuscript. All authors read and approved the final manuscript.

## Authors’ information

Brian L Ellis, Matthew L Hirsch, Jenny C Barkerm, denotes co-first authorship.
